# Pectin- Derived Acidic Oligosaccharides Improve the Outcome of *Pseudomonas aeruginosa* Lung Infection in C57BL/6 Mice

**DOI:** 10.1371/journal.pone.0139686

**Published:** 2015-11-23

**Authors:** Henry Bernard, Jean-Luc Desseyn, Frédéric Gottrand, Bernd Stahl, Nana Bartke, Marie-Odile Husson

**Affiliations:** 1 LIRIC UMR 995 Inserm; Université de Lille; CHRU de Lille, Faculté de Médecine, Place de Verdun, F-59045, Lille cedex, France; 2 Nutricia Research, Uppsalalaan 12, 3584 CT Utrecht, The Netherlands; University of North Dakota, UNITED STATES

## Abstract

The administration of prebiotics as oligosaccharides (OS), by acting on intestinal microbiota, could modulate the immune and inflammatory response and represent a new strategy to improve the outcome of bacterial infection. The aim of this study was to determine whether pectin-derived acidic oligosaccharides (pAOS) could modulate the outcome of pulmonary *P*. *aeruginosa* (PA) infection in C57BL/6 mice, which develop a Th1 response to PA lung infection. Mice were randomized for 5 weeks to consume a control or a 5% pAOS diet and chronically infected by PA. Resistance to a second PA infection was also analyzed by reinfecting the surviving mice 2 weeks after the first infection. Compared with control mice, mice fed pAOS had reduced mortality (*P*<0.05). This improvement correlated with a better control of the inflammatory response with a lower neutrophil count on day 1 (*P*<0.05), a sustained neutrophil and macrophage recruitment on days 2 and 3 (*P*<0.01) a greater and sustained IL-10 release in lung (*P*<0.05) and a reduction of the Th1 response and M1 activation with a lower IFN-γ/IL-4 (*P*<0.01) and *nos2/arg1* (*P*<0.05) ratios. These results coincided with a modulation of the intestinal microbiota as shown by an increased butyric acid concentration in feces (*P*<0.05). Moreover, pAOS decreased the bacterial load (*P*<0.01) in mice reinfected 2 weeks after the first infection, suggesting that pAOS could reduce pulmonary exacerbations. In conclusion, pAOS improved the outcome of PA infection in C57BL/6 mice by modulating the intestinal microbiota and the inflammatory and immune responses.

## Introduction

Dietary oligosaccharides (OSs) such as long-chain fructo-oligosaccharides (lcFOS) and short-chain galacto-oligosaccharides (scGOS) are carbohydrate molecules with prebiotic properties [1]. Resistant to digestion in the upper gastrointestinal tract, these substances reach the colon where they stimulate the growth of bifidobacteria and lactobacilli [2,3] which are known to induce Th polarization [4,5] and regulatory T cell (Treg) responses [6]. OSs are also degraded by intestinal microbiota into short-chain fatty acids (SCFAs) such as butyric acid and propionic acid, which also regulate several leukocyte functions and promote T cell differentiation and Treg cell functions [7–9]. The immune properties of OS have been demonstrated to decrease Th2-dependant response expressed in allergic disease [10,11] or to increase the Th1-dependent response involved in the virus-induced delayed-type hypersensitivity response [12,13]. From a recent meta-analysis, they could also decrease the rate of viral and bacterial infections requiring antibiotics treatment in infants and children 0–24 months [14]. Thus, the administration of prebiotics, by acting on the immunity through their action on intestinal microbiota, could represent a new strategy for the prevention of acute or chronic infectious diseases and the reduction of antibiotic treatment.

*Pseudomonas aeruginosa* (PA), a Gram-negative environmental bacilli is an opportunistic pathogen that can cause pneumonia in immune compromised patients and chronic pulmonary infections in people with cystic fibrosis (CF) and chronic obstructive pulmonary disease. In each of these situations, PA infection is characterized with an excessive recruitment of neutrophils, a significant inflammation in the lung parenchyma which leads to tissue damage, and an ineffective immune response which is unable to clear the bacteria [15,16]. PA infections are severe and lead regularly to significant morbidity and mortality [15].

The aim of this study was to assess the effects of pAOS intervention in C57BL/6 mice known to develop a severe pulmonary infection with an excessive pulmonary inflammatory and a Th1 immune responses [17]. These mice were chronically infected with PA after 5 weeks of pAOS administration, and the effects of pAOS were assessed by measuring the survival of mice, pulmonary bacterial clearance, concentrations of inflammatory and immune markers, and expression of *nos2* and *arg1* genes which indicate M1 and M2 macrophage activation, respectively [18]. These effects were compared with the action of pAOS on intestinal microbiota by measuring the fecal SCFA concentration. Resistance to a second PA infection was then analyzed by reinfecting the surviving mice 2 weeks after the first infection and by measuring the pulmonary bacterial load.

## Materials and Methods

### Animals and diets

Three-week-old male C57BL/6 mice purchased from Harlan Laboratories (Gannat, France) were randomized into two groups and fed either a diet enriched in 5% pAOS extracted from citrus or a control diet containing 5% cellulose instead of pAOS (Nutricia Research, The Netherlands) for 5 weeks before PA infection. Both diets had the same formulation as that of the control diet (based on AIN-93G [19] in terms of the ratios of energy, protein, fat, mineral, micronutrient, and vitamin contents. At the date of the experiments of this study (September 2011-June 2013), agreement by an ethical office for protocols using live animals were not mandatory under French law. However, all experiments on mice in this study were conducted in accordance with the French Guide for the Care and Use of Laboratory Animals and the Guidelines of the European Union in the animal facility of our University (Dhure/IMPRT/IFR114) which possesses the approval number A59-35015. Furthermore, the authors of the study H. Bernard and J-L. Desseyn and only the staff of the animal facility, who have personal licenses from the French Veterinary Service, infected and sacrificed the mice. Animals were surveyed during the first 8 hours of infection for the criteria bristly hair, arched back, and sunken eyes which were matched 1, and tachycardia and stillness which were matched 2. When the score was superior to 3, mice were killed by a peritoneal injection of pentobarbital (300 μL of a 30μg/mL solution) followed by cervical dislocation. Within the two diet groups, four groups of 15 mice were used for studying survival, five to six groups of 10 mice were used for studying bacterial and immune parameters in the lungs and five to six groups of 10 mice for measuring inflammatory parameters in the bronchoalveolar lavage fluid (BALF).

### SCFAs contents

Fresh feces specimen from three to four mice fed either pAOS or control diet and housed in separate cages were collected just before the beginning of diet intervention (day 0) and after 1, 2, 3 and 5 weeks of diet intervention, and immediately stored at –80°C. SCFA concentrations were measured concomitantly and in triplicate by solid-phase micro-extraction headspace gas chromatography with flame ionization detection (SPME-HS-GC-FID) as described in detail previously [20].

### Lung infection and BALF collection

Lung infection was initialized by an endotracheal injection of 5x10^5^ PA strain PAO1 or 0.9% saline solution for the control group entrapped in agar beads as described previously [21]. Lungs for bacterial counts, interferon-γ (IFN-γ) and interleukin IL-4 contents and RT-qPCR, and BALF for cell count and dosage of keratinocyte chemoattractant (KC), tumour necrosis factor-alpha (TNF- α), and IL-10 were collected as described in detail previously [21]. Surviving animals were reinfected following the same protocol 2 weeks after the first infection, and sacrificed on days 1–3 for bacterial counts in their lungs.

### RT-qPCR

Total RNA from frozen lungs and qRT-PCR in duplicate for *nos2* and *arg1* genes and normalized against the expression of β*-*actin were performed as described in detail previously [20]. The results were expressed as fold changes in expression on each day for infected mice compared with uninfected mice.

### Cytokine and chemokine contents

The concentrations of cytokines and chemokines were measured using an enzyme-linked immunosorbent assay kit (R&D Systems, Lille and CliniSciences, Montrouge, France).

### Statistical methods

The SCFA concentrations expressed as means and bacterial load, inflammatory and immune parameters expressed as medians were compared over time within the pAOS and control groups to assess the changes with time and between the two groups at the different times using the Mann–Whitney *U* test to assess the diet effect. The statistical software GraphPad Prism 5.0 was used. *P*<0.05 was considered significant. Cumulative survival rates were compared using the log-rank test.

## Results

### SCFA concentrations in feces

The concentrations of SCFAs differed between the mice fed the control diet and those fed the pAOS diet from day 7 for the shortest SCFA acetic acid, day 14 for propionic and butyric acids, and day 21 for the longest SCFAs iso-valeric acid and valeric acid. These changes indicated a modulation of the intestinal microbiota (Table 1). The concentrations of acetic acid, butyric acid, valeric acid, and isovaleric acid remained significantly higher (*P*<0.05) in the faeces of mice fed the pAOS diet compared with the control group after 5 weeks of dietary intervention and just before infection.

**Table 1:** concentrations of SCFAs according diets. Concentrations (μg/100mg) of acetic acid (C2:0), propionic acid (C3:0), isobutyric acid (C4:i), butyric acid (C4:0), isovaleric acid (C5:0i) and valeric acid (C5) in feces of mice fed the control or pAOS diet at different times during the dietary intervention.

	day 0	day 7	day 14	day 21	day 35					
SCFA	control	pAOS	control	pAOS	control	pAOS	control	pAOS	control	pAOS
**C2:0**	55±134	624±174	65.2±112	438±60*	175±64.4	474±61*	115±15.6	316±31*	232±42.5	461±39*
**C3:0**	30.1±8.8	40.3±12.4	7.7±3	10.9±2.6	20.7±1.1	31.9±7.6*	10.6±2.2	28.7±11*	6±3.5	9.2±1.5
**C4:i**	0.5±0.2	0.9±0.5	0.7±0.3	0.7±0.1	2.4±0.5	3.1±1.3	1.1±0.2	1.9±0.9*	0.5±0.3	0.9±0.1
**C4:0**	4.9±2.1	9.7±5.3	0.8±0.3	1.6±0.7	0.1±0.1	0.4±0.1*	0.9±0.7	6.5±4.8*	0.5±0.4	3.5±0.3*
**C5:i**	0.9±0.3	1.3±0.5	1.1±0.4	1.3±0.4	3.8±1	3.9±1.4	2.1±0.3	3.2±1*	0.8±0.4	2.0±0.2*
**C5:0**	1.6±0.5	1.6±0.5	0.8±0.5	0.7±0.2	0.2±0.2	0.6±0.4	0.3±0.2	3.2±2.5*	0.2±0.1	0.9±0.1*

Data were analysed using the Mann-Whitney*U* test.

*P<0.05.

### First PA infection

**Fig 1:**
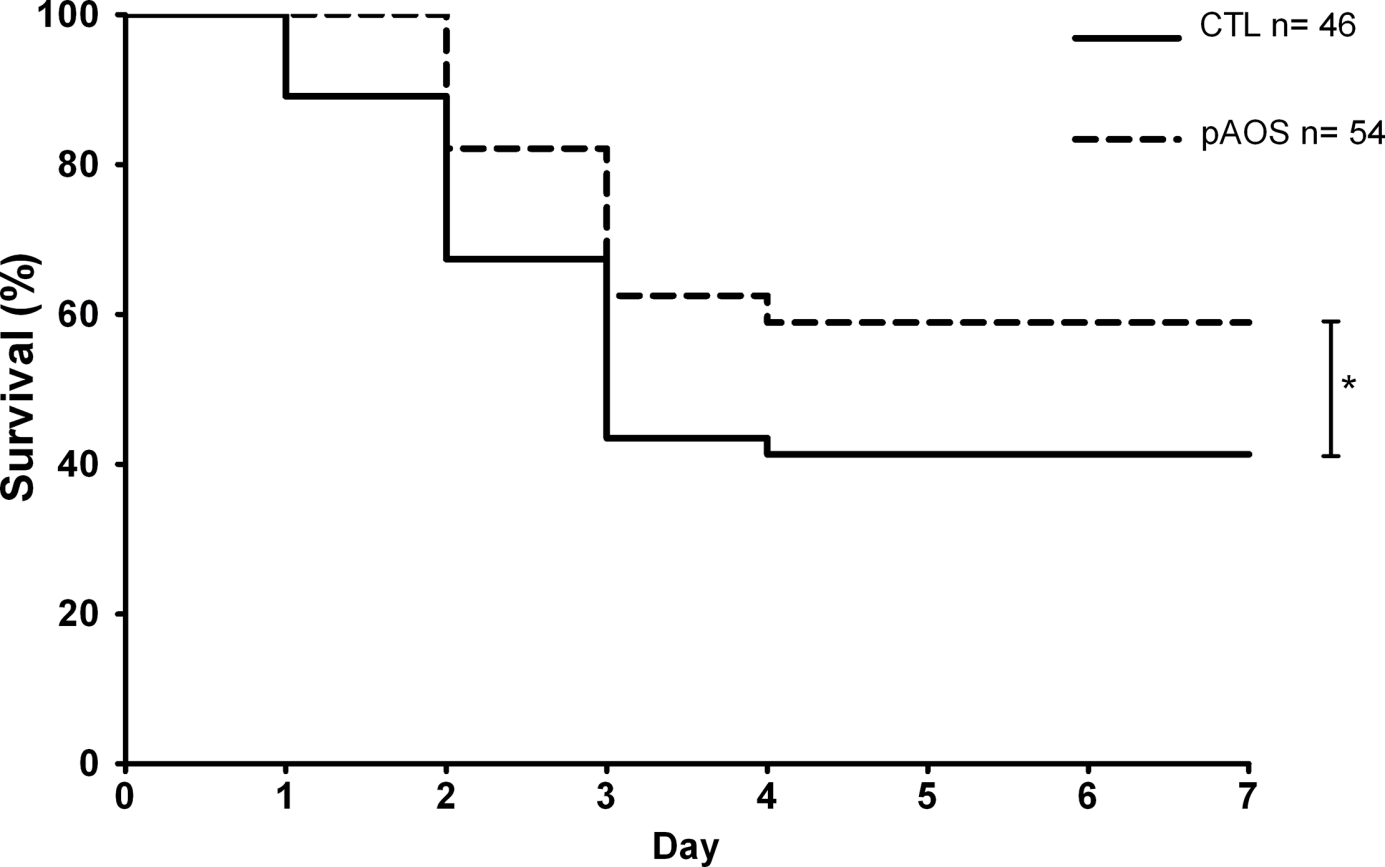
Survival rates according to diets. Survival rates after PA infection in mice fed the pAOS or control diet. The p-value is based on the log-rank test. *, *P*<0.05.

The percentage of surviving mice was significantly higher (*P*<0.05) in mice fed the pAOS diet (58%) than in mice fed the control diet (41%) (Fig 1).

**Fig 2:**
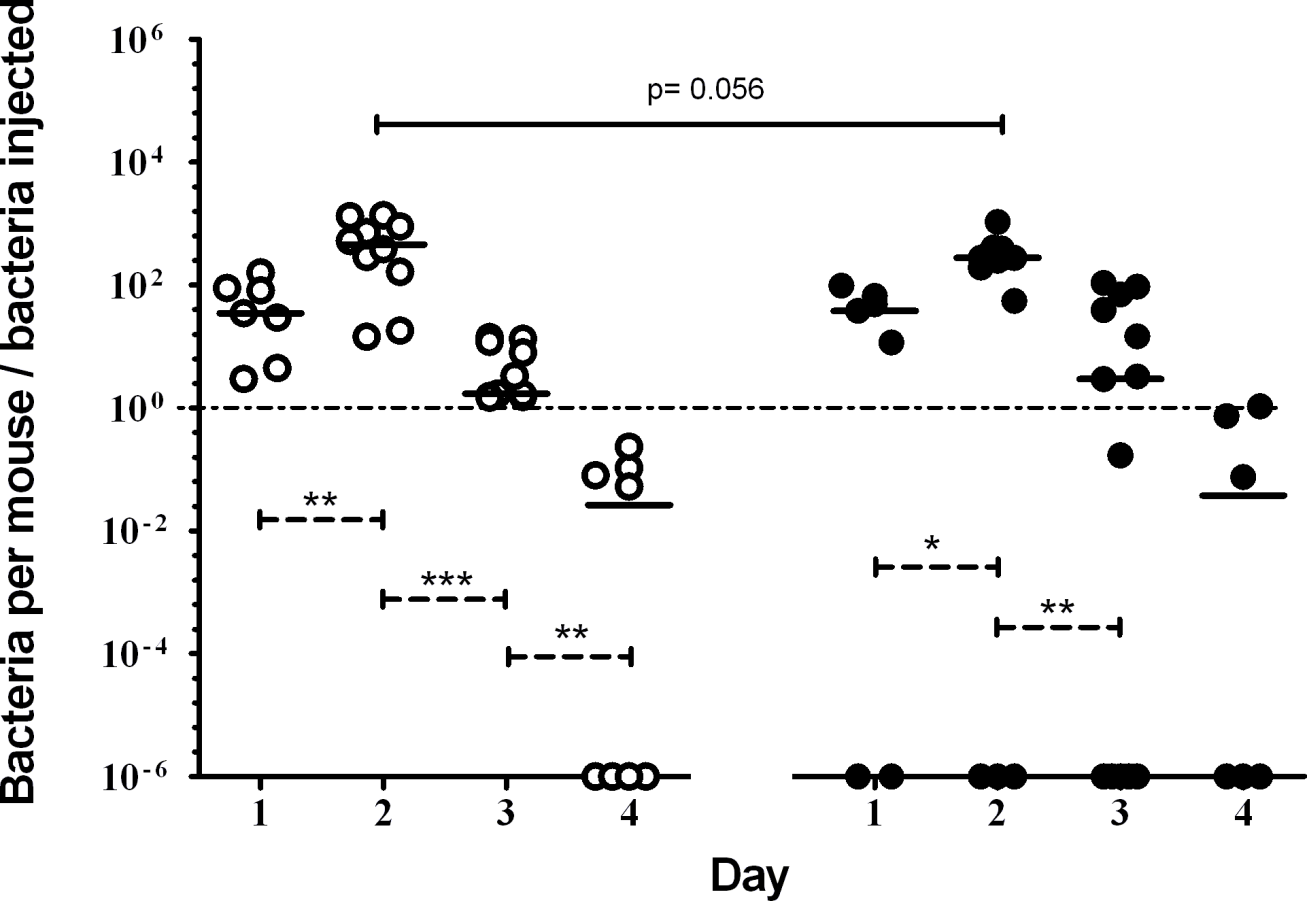
Bacterial load in lungs according to diets. Bacterial load in lungs assessed as the ratio of the number of bacteria recovered from each mouse and the number of bacteria injected after a first PA infection. Each open circle represents one mouse fed the control diet; each black circle represents one mouse fed the pAOS diet. Bars represent median values. Dotted lines indicate differences between each day of infection within each dietary mice group; solid lines indicate differences between the pAOS and control mice groups on the same day; The p-values are based on the MannWhitney U test. *, *P*<0.05; **, *P*<0.01.

Mice fed the pAOS diet died between days 2 and 4, whereas mice fed the control diet started to die earlier and died between days 1 and 4. No bacteria were observed from the culture of the lungs of 2, 3 and 4 mice fed the pAOS diet on days 1, 2 and 3, respectively. By contrast, bacterial cultures from mice fed the control diet indicated infection in all mice during the first 3 days. However pAOS intervention did not decrease significantly the bacterial load (Fig 2).

### Inflammatory parameters after the first infection

**Fig 3:**
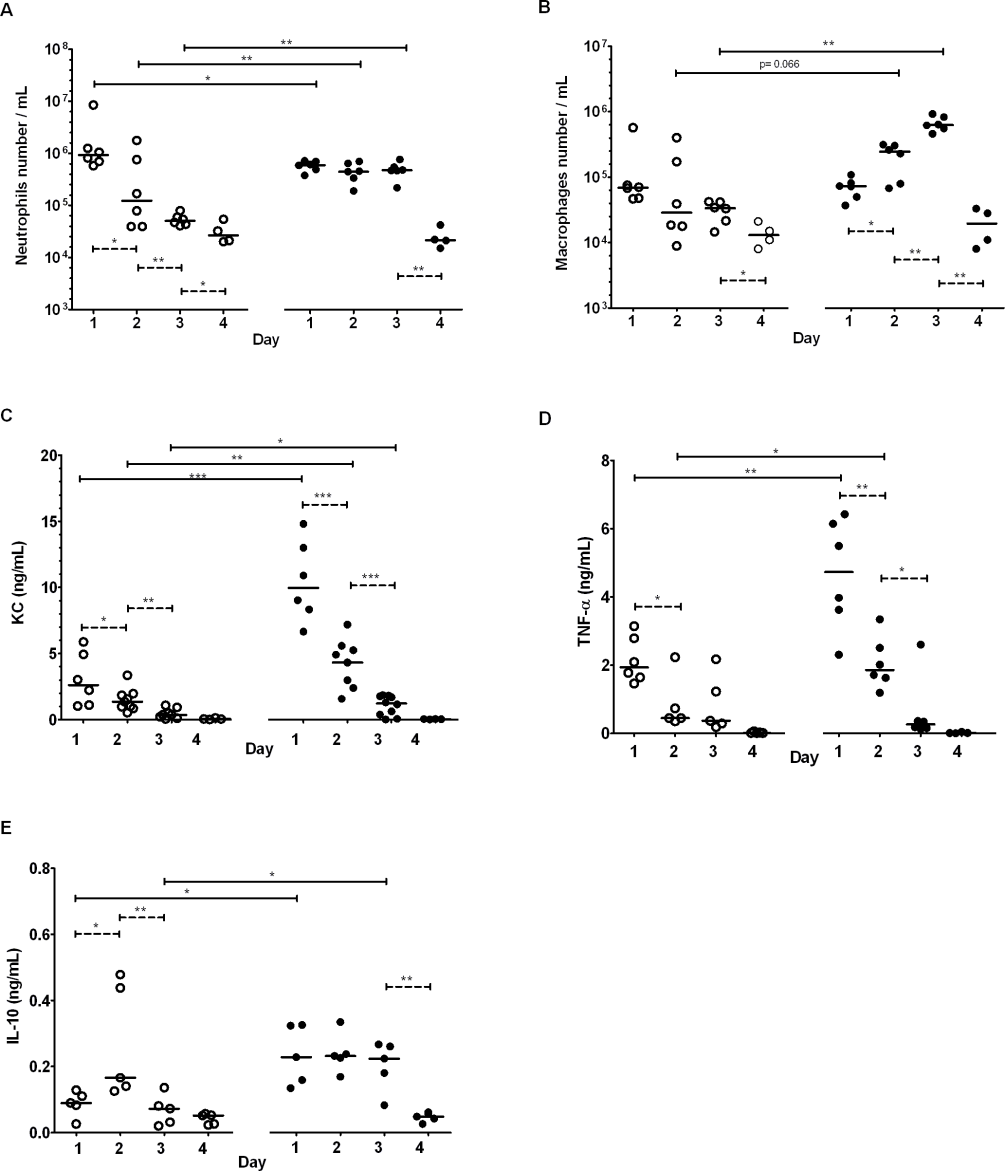
Inflammatory response according to diets. Inflammatory response assessed in the BALF of the pAOS and control mice during the first 4 days of PA infection. *A*, number of neutrophils. *B*, number of macrophages. *C*, KC concentration. *D*, TNF-α concentration. *E*, IL-10 concentration. Each open circle represents one mouse fed the control diet; each black circle represents one mouse fed the pAOS diet. Bars represent the median values. Dotted lines indicate differences between each day of infection within each dietary mice group; solid lines indicate differences between the pAOS and control mice groups on the same day. the p-values are based on the Mann-Whitnney U test. *, *P*<0.05; **, *P*<0.01.

The number of neutrophils was significantly lower in the pAOS-fed mice compared with the control mice on day 1 (*P*<0.05; Fig 3A) and remained stable during the first 3 days before decreasing significantly on day 4 (*P*<0.01) in the pAOS-fed group. By contrast, in mice fed the control diet, neutrophil count decreased significantly from day 1 to day 2 (*P*<0.05) and to other days (*P*<0.01). The number of macrophages was similar in the two groups of mice on day 1 (Fig 3B), but increased significantly on days 2 (*P*<0.05) and 3 (*P*<0.01) in mice fed the pAOS diet while it remained stable during the first 3 days in mice fed the control diet. The concentrations of the inflammatory chemokine KC (Fig 3C) and TNF-α (Fig 3D) were higher on day 1 (*P*<0.001 and *P*<0.01), day 2 (*P*<0.01 and *P*<0.05), respectively), and day 3 (*P*<0.01 for both) in pAOS-fed mice compared with control mice. IL-10 release was greater (*P*<0.05) on day 1, remained stable during the first 3 days of infection and was higher on day 3 in the pAOS group compared with the control group (Fig 3E).

### Immune response after the first PA infection

**Fig 4:**
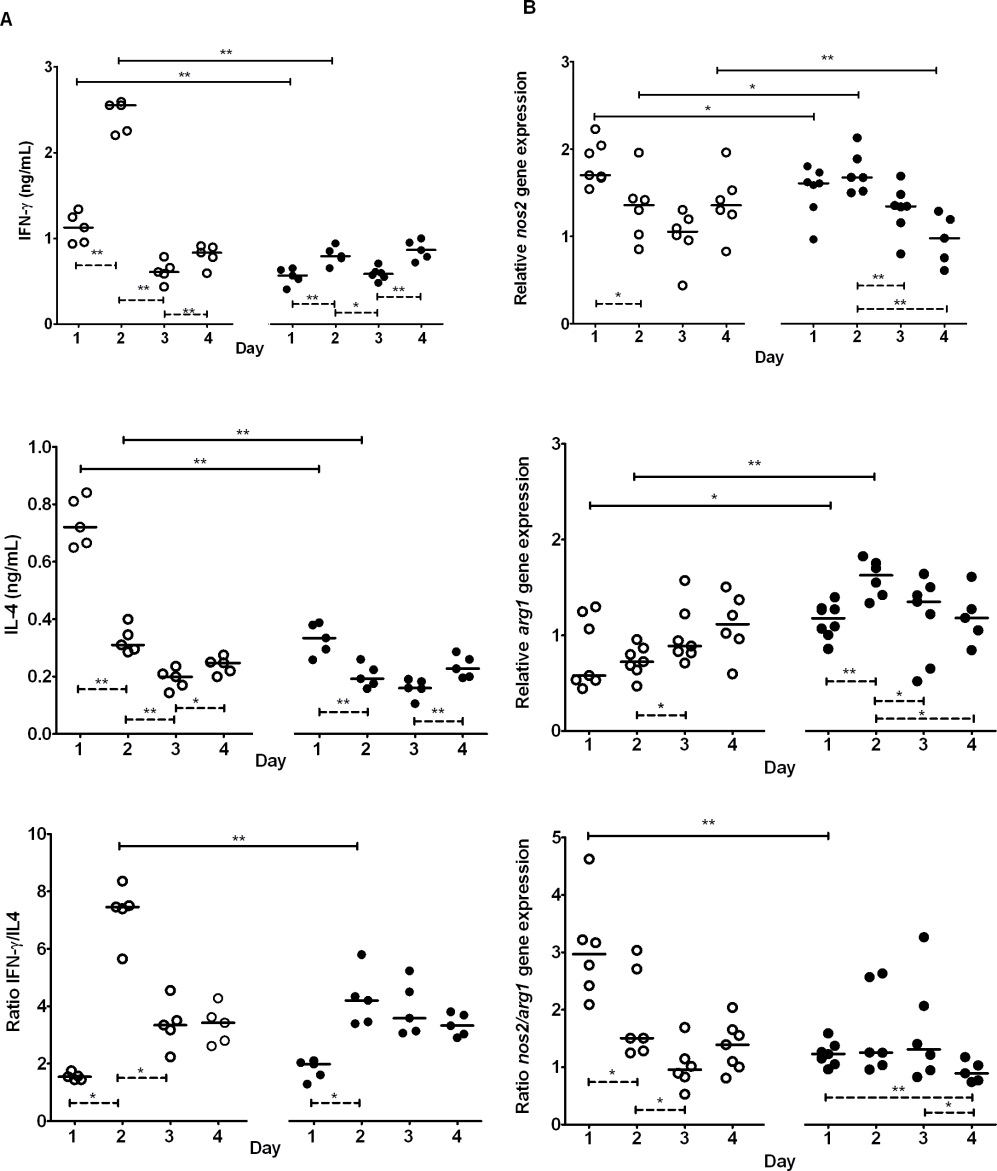
Immune response according to diets. Immune response assessed in the lungs of mice fed the pAOS or control diet during the first 4 days after PA infection. *A*, IFN-γ IL-4 concentrations and IFN- γ/IL-4 ratio. *B*, *nos2*, *arg1*, and *nos2/arg1* mRNA levels. Each open circle represents one mouse fed control diet and each black circle represents one mouse fed pAOS diet. Bars represent median values. Lines indicate differences between each day of infection within each dietary mice group or between the pAOS and control mice groups on the same day of infection.; The p-values are based on the Mann-Whitney U test. *, *P*<0.05; **, *P*<0.01

The kinetics of the release of IFN- γ (Th1) and IL-4 (Th2), and the ratio IFN-γ/IL-4 in infected lung tissues were similar in the pAOS and control mice (Fig 4A). However, the IFN-γ and IL-4 rates were significantly lower on day 1 (*P*<0.01) and day 2 (*P*<0.01) in the pAOS group compared with the control group. These variations led to a smaller increase of the ratio IFN-γ /IL-4 on day 2 in the pAOS group (*P*<0.01) compared with the control group, indicating a reduction in the Th1 immune response.

The *nos2* mRNA level (M1) was lower on day 1 (*P*<0.05) and remained stable during the first 2 days before decreasing significantly (*P*<0.01) on days 3 and 4 in pAOS-fed mice compared with control mice, in which it decreased earlier on day 2 (Fig 4B). By contrast, the *arg1* mRNA level (M2) was higher on days 1 (*P*<0.05) and 2 (*P*<0.01) in the pAOS group than in the control group. These variations led to a lower *nos2/arg1* ratio in the pAOS group compared with the control group on day 1 (*P*<0.01), indicating a reduction in M1 activation.

### Bacterial pulmonary clearance after the second PA infection

**Fig 5:**
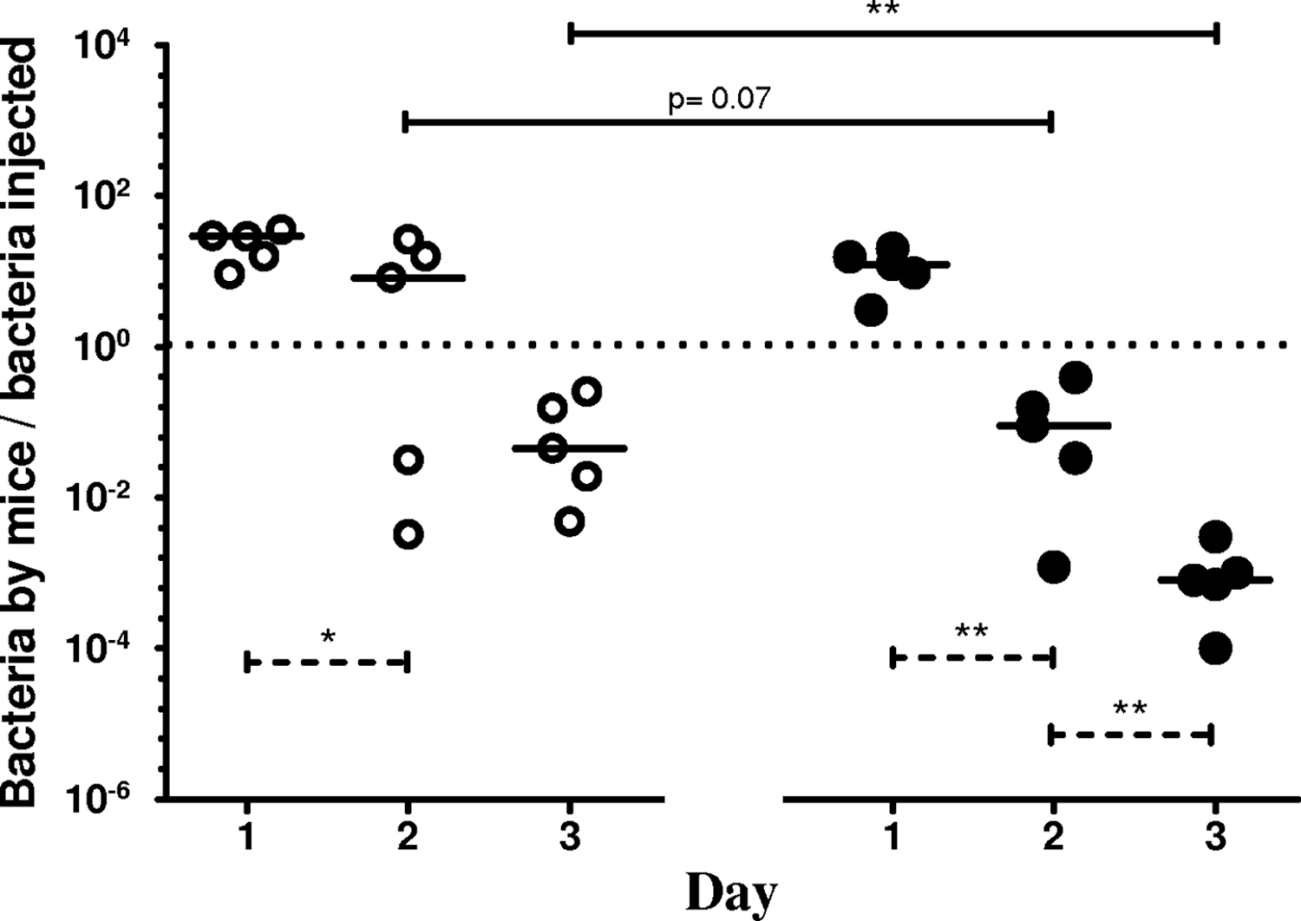
Bacterial load after a second infection according to diets. Bacterial load in lungs assessed as the ratio of the number of bacteria recovered in each mouse and the number of bacteria injected, after the second PA infection. Each open circle represents one mouse fed control diet, and each black circle represents one mouse fed pAOS diet. Bars represent median values. Dotted lines indicate differences between each day of infection within each dietary mice group; solid lines indicate differences between the pAOS and control mice groups on the same day. The p-values are based on the Mann-Whitney U test. *, *P*<0.05; **, *P*<0.01.

After the second infection, no reinfected mice died in either group. The number of bacteria decreased rapidly after the second infection in the two groups but was significantly lower on day 3 in pAOS-fed mice compared with control mice (*P*<0.01; Fig 5).

## Discussion

Our results show that oral administration of pAOS improved the outcomes of PA pulmonary infection in C57BL/6 mice by reducing the mortality rate after a first infection and the bacterial load after the second infection. During the first infection, this improvement correlated with a progressive recruitment of phagocytic cells, as shown by the lower neutrophil count on day 1, a stable neutrophil count during the two following days, and an increased macrophage count in the pAOS-fed mice. These two changes coincided with higher concentrations of the inflammatory chemokine KC and cytokine TNF-α in pAOS-fed mice. pAOS administration also reduced the Th1 immune response and M1 activation, suggesting a limited production of cytotoxic lymphocyte T and cytotoxic nitric oxide products, respectively. These results along with the higher and sustained IL-10 release observed in pAOS-fed mice suggest that pAOS improved the outcome of PA infection by reducing inflammation and limiting tissue damage.

The effect of pAOS was associated to an increase of butyric acid concentration in the feces of C57BL/6 mice. Butyric acid is a product of carbohydrate fermentation by many intestinal bacterial species [22,23]. This major metabolite of intestinal bacterial fermentation possesses local and systemic anti-inflammatory and immune-modulating properties after crossing the intestinal barrier. It can influence the recruitment of circulating leukocytes to inflammatory sites, increase the antimicrobial activity of macrophages, interfere with T cell differentiation, and reduce induced Th1 cytokines [24]. A main property of butyrate is its capacity to induce IL-10 production by Treg cells [25]. This property is important because IL-10 can counteract the effects of inflammatory cytokines such as TNF-α and KC, and can act as a modulator of disrupted balance of T-cell responses. Thus, the beneficial effects of pAOS may arise from their actions on intestinal microbiota, which increase butyrate and IL-10 production, both of which act in the resolution of inflammation.

These results complement the beneficial effects of oral pAOS intervention on the outcome of PA infection observed in BALB/c mice which are known to exhibit another immune and inflammatory response [20]. These mice are known to exhibit a low inflammatory response and a Th2 immune response, both mechanisms leading to a high bacterial load in their lung [17,26]. In this model, pAOS did not decrease mortality and inflammation, but induced a shift from the Th2 to Th1 immune response and M1 activation that reduced the bacterial pulmonary load from the first infection. These results were also correlated with the pAOS action on intestinal microbiota with an increase of fecal butyric acid concentration, and with the increase of IL-10 release in the lung [20]. Thus, pAOS could improve the outcome of PA infection in BALB/c as in C57BL/6 mice, even if both mouse strains develop different immune and inflammatory responses to PA infection. Although regression analysis and Spearman’s rank correlation could not be computed between butyric acid levels and cytokine levels, the higher butyric acid production in the pAOS group, which was found in the two models, likely explains the immune and anti- inflammatory properties of pAOS. Moreover, a recent study reported that intragastric injection of butyrate reduced inflammation and attenuated lipopolysaccharide-induced acute lung injury in mice [27]. All results are of interest in CF patients, for whom predominant Th2 and Th1 responses are observed [28]. Because Th2 immune response is anti-inflammatory and has only a low phagocytic activity, Th2 immune profile correlates with excessive pulmonary exacerbations and has poor prognosis [29]. At the contrary, a predominant Th1 immune response coincides with a high phagocytic activity that reduces the number of PA in the lung, but increases the pulmonary inflammation [30]. Besides, the bacterial species, that degrade OS and produce butyric acid as Bifidobacteria, *Faecalibacterium prausnitzii*, and Clostridium cluster XIVa [31,32], could be reduced after antibiotic treatment [33] and are deficient in CF patients [34,35].

These results are also in agreement with findings demonstrating the influence of indigestible OS on the systemic immune and inflammatory responses through their prebiotic activity [10–13] and their influence for prevention of acute infectious diseases [14].

pAOS could also act independently of their action on intestinal bacteria. A recent study showed that different indigestible OS can act directly as TLR-4 ligands on intestinal epithelial cells and thus directly influence the immune response [36]. Besides, OS could also cross the intestinal barrier [37] and act directly on immune cells as recently demonstrated [38].

In conclusion, pAOS improved the outcomes of PA infection in C57BL/6 mice by reducing the mortality at the first infection and accelerating bacterial clearance at the second infection that could be beneficial for reducing pulmonary exacerbation. These improvements were associated with an increase of the fecal concentration of butyric acid suggesting their action on intestinal microbiota. These results suggest that the oral administration of pAOS could reduce the severity of PA infection and represent a non-aggressive alternative to reduce antibiotic treatment.

## Abbreviations

ARG1: Arginase1
AIN: American Institute of Nutrition
BALF: Bronchoalveolar lavage fluid
CF: Cystic fibrosis
scGOS: Short-chain galacto-oligosaccharides
OS: Oligosaccharides
NOS2: Nitric oxyde synthase 2
PA: *Pseudomonas aeruginosa*
pAOS: Pectin derived acidic oligosaccharides
SCFA: Short-chain fatty acids
lcFOS: Long-chain fructo-oligosaccharides
SPME-HS-GC-FID: Solid-phase micro-extraction headspace gas chromatography with flame ionization detection
Treg: Regulatory T cells
